# Chinese non-psychiatric hospital doctors’ attitudes toward management of psychological/psychiatric problems

**DOI:** 10.1186/s12913-017-2521-8

**Published:** 2017-08-22

**Authors:** Jun Wang, Qun Wang, Inoka Wimalaratne, David Benjamin Menkes, Xiaoping Wang

**Affiliations:** 1 Mental Health Institute of the Second Xiangya Hospital, Central South University, Changsha, Hunan 410011 People’s Republic of China; 2 National Clinical Research Center on Mental Disorders & National Technology Institute on Mental Disorders, Changsha, Hunan 410011 People’s Republic of China; 3 Hunan Key Laboratory of Psychiatry and Mental Health, Changsha, Hunan 410011 People’s Republic of China; 4 Waikato District Health Board, University of Auckland, Hamilton, 3240 New Zealand

**Keywords:** Doctors’ attitudes, Psychological problems, Psychiatric disorder, Consultation-liaison psychiatric services, General hospitals

## Abstract

**Background:**

Psychiatric comorbidities are common among patients treated for physical disorders. Attitudes of non-psychiatric doctors toward psychological/psychiatric problems have significant implications for care provision in the general hospital setting. Our objective was to investigate non-psychiatric doctors’ attitudes in China.

**Method:**

An anonymous online questionnaire pertaining to relevant attitudes was distributed to Chinese hospital-based non-psychiatric doctors using a mobile App.

**Results:**

A total of 306 non-psychiatric doctors in China voluntarily completed the questionnaire. All but two (99.3%) respondents agreed with the importance of psychological factors underlying physical illness and 85.6% agreed they had a high degree of responsibility for management of patients’ emotional problems. Most respondents endorsed routine assessment of patients’ psychological factors and were willing to consider psychiatric referrals for patients in need; despite 52.0% believing that mental health care by general hospital doctors was impractical. Almost all respondents welcomed more contact with psychiatric services and indicated a need for more time and professional help to manage psychological issues. Respondents’ demographic characteristics and vocational status had some influence on attitudes; female doctors were more likely and surgeons less likely to consider psychological assessment and emotional care for patients with physical illness. More doctors working in hospitals with established consultation-liaison psychiatric services did not feel responsible for their patients’ emotional care (17.7% vs. 6.6%, *P* = 0.012).

**Conclusions:**

Our pilot survey demonstrates a potential generally positive attitude toward management of patients’ psychological problems and an urgent need for more time and specialist support for non-psychiatric doctors in China.

## Background

Studies have indicated complex relationships between mental and physical illnesses [1, 2]. Individuals with physical illness have a higher prevalence of mental disorder compared with the general population [3], while psychiatric illnesses are known risk factors for the occurrence, development and poor outcome of, for example, cardiovascular disease, diabetes and cancer [4, 5].

It is a truism that there is no mental health without physical health [6]; likewise, there is no physical health without mental health either [7]. As defined by the World Health Organization, health is “a state of complete physical, mental, and social well-being and not merely the absence of disease or infirmity” [8]. Moreover, psychological problems are sometimes more disabling than physical illnesses. Despite their high prevalence, comorbid psychiatric disorders are often neglected in general hospital settings [9].

Patients often lack knowledge and awareness of mental illness [10]. Common psychological problems such as depression and anxiety are often accompanied by somatic complaints including pain, fatigue, malaise, dizziness, dyspepsia, etc. [11–14]. Patients lacking insight into psychological problems [10] typically consult general practitioners or physical medicine specialists regarding such somatic symptoms. Doctors often struggle to effectively manage such patients for several reasons, including lack of time and necessary skills. In addition, stigma caused by prevailing bias of the public towards mental illness [15] may hinder patients from consulting a psychologist or psychiatrist. Likewise, stigma can discourage doctors from considering psychiatric referral. Consequently, patients with such problems often seek help from non-psychiatric doctors for a long time before visiting a mental health practitioner.

Consultation-liaison psychiatry (CLP) [16], initially launched in America and subsequently adopted worldwide, was intended to resolve this dilemma. CLP in general hospitals provides mental health assessment and treatment for patients with psychological problems, as well as training medical students and non-psychiatric doctors to recognize and manage psychiatric comorbidities [16]. Ideally, non-psychiatric doctors are able to improve their skills in management of patients’ psychological problems with support from CLP. Even when problems extend beyond their scope and time constraints, doctors can still provide appropriate care for patients by referring them to psychiatry. In many Western countries, CLP is now an established subspecialty of psychiatry; however, the situation in China is less developed [17].

A recent study indicated that the overall practice of CLP in China remains basic despite its foundation decades ago [17]. Until recently, there has been limited collaboration between the departments of psychiatry and other specialties, and rates of psychiatric referral and consultation for general hospital patients are still very low [17]. The ability to identify co-morbidity of common psychiatric disorders remains unsatisfactory among a majority of non-psychiatric doctors [18, 19]. For example, the Chinese Ministry of Health launched *China’s working plan for mental health (2002–2010)* in 2003 [20], aiming to enhance the recognition of depression in general hospitals. However, this failed to achieve the expected goals [17]. Since human social behavior is influenced by attitudes to a large extent [21], it is important to investigate doctors’ attitudes toward psychiatry and the management of patients’ psychological problems in order to better understand and improve the situation in China.

Several surveys regarding doctors’ management of psychological problems have been conducted in Western countries, and results cited as evidence of barriers to the development of CLP [22, 23]. However, similar studies in China are rare, despite the reported high prevalence of psychiatric disorders among patients and low recognition rates by general hospital doctors [18, 19, 24–26]. On the other hand, there has been substantial development in mental health services in China in recent decades. A particular milestone, Chinese Mental Health Law, became effective in 2013 after more than two decades of drafting, consultation and revision [27]. This law aims to promote and regulate public mental health services, and is expected to have a positive influence on public attitudes toward psychiatric disorders and mental health in general. In order to better understand current attitudes toward management of psychological problems among Chinese non-psychiatric doctors, we carried out an online survey.

## Methods

### Participants

An anonymous online survey was conducted during December 2015 and January 2016. General hospital non-psychiatric doctors of Chinese ethnicity were invited to take part. Participation was voluntary and all participants provided online informed consent. The study protocol was approved by the Ethics Committees of the Second Xiangya Hospital, Central South University.

### Instrument

The questionnaire was translated (and checked with back-translation) from an English version developed by Dr. Inoka Wimalaratne as part of an ongoing international study. It is based in part on instruments used in previous studies [22, 23, 28] and comprises 22 questions pertaining to general hospital doctors’ attitudes toward the assessment, referral and management of psychological problems, as detailed in Table 1. In particular, respondents were asked to identify reasons when choosing not to consider or make a psychiatric referral. We also collected non-identifying demographic information including vocational status, gender, age band, educational level, current job, professional title, and whether there is a functioning CLP service within the respondent’s hospital, as the second type described by Ji and Ye [17]. The questionnaire required 10–15 min to complete.

**Table 1:** Demographic characteristics of respondents

Categories		Number (n)	Percentage (%)
Gender	Male	154	50.3
	Female	152	49.7
Age band	Less than 30	127	41.5
	30–39	118	38.6
	40–49	49	16.0
	50 and above	12	3.9
Educational level	Bachelor and below	133	43.5
	Master and above	173	56.5
Specialty	Physician	163	53.3
	Surgeon	75	24.5
	Gynecologist or Obstetrician	24	7.8
	Pediatrician	21	6.9
	Geriatric Doctor	8	2.6
	Accident and Emergency Doctor	15	4.9
Title	Junior	144	47.1
	Middle	103	33.7
	Senior	59	19.3
Hospital level	The first-level hospital	25	8.2
	The second-level hospital	58	19.0
	Tertiary hospital	223	72.9
CLP service	Yes	215	70.3
	No	91	29.7

*CLP* consultation-liaison psychiatry

### Procedure

The study was carried out by means of a network platform, WenJuanWang (https://www.wenjuan.com/) and a mobile application, WeChat (http://www.wechat.com/). WenJuanWang is the largest free online survey platform in China, which is similar to SurveyMonkey in USA (https://www.surveymonkey.com/). WeChat is one of the largest standalone messaging applications in China, and is available on Android, iPhone, BlackBerry, Windows and Symbian phones. WeChat users can freely and easily distribute information in several ways. The questionnaire was firstly distributed to doctors we knew to be available, and receivers were encouraged to distribute it to other relevant doctors.

An introduction briefly explained the survey aim; online informed consent was obtained before the start. Participants were asked to complete the questionnaire without consulting others. To exclude participants beyond the scope of our survey, every participant was asked to state his/her present occupational status before answering the questions. Three options were given: “Psychiatrist/Psychologist”, “Non-psychiatrist/Non-Psychologist doctor”, and “Others (such as nurse, clinical laboratory personnel, etc.)”. Only participants choosing the 2nd option were able to proceed further. A restriction was set to avoid repeating the survey by same person, that is, each WeChat account allowed participation on a single occasion only.

### Statistical analysis

Data were collected by WenJuanWang and analyzed using IBM SPSS Statistics, Version 22 (IBM, New York, USA). All statistical tests were two-tailed, and an alpha of 0.05 was used. Descriptive statistics were used to record the percentage of agreement with each question. Chi-square or Fisher’s exact tests were used to analyze differences between 2 subgroups divided by different demographic characteristics and vocational status when appropriate. When there were 3 or more subgroups, mutiple subgroup analyses were conducted using binary logistic regression, by setting repondents’ attitudes as dependent variable, and subgroup as categorical independent variable. To minimize risk of type I error caused by small sample size, sets with *n* < 30 were combined with a neighboring set. For example, there were only 12 respondents aged 50 and above, and thus this age band was combined with its neighboring age band 40 to 49 into a new set of 40 and above. Similarly, we combined pediatricians, gynecologists, obstetricians, geriatric doctors, and accident and emergency doctors into a new set of “Other doctors”, for statistical comparison with physicians and surgeons.

## Results

### Sample description

Three hundred twelve doctors voluntarily participated and completed the questionnaire. Six were excluded from analysis, 5 were postgraduate students and 1 was working in the United States at that time. Of the remaining 306 respondents, 154 (50.3%) were male, 127 (41.5%) aged less than 30 years, and 173 (56.5%) held a master’s degree or above (Note: different from many other countries, the master degree is not a prerequisite for a person to be a doctor in China). More than two thirds of respondents were employed in tertiary hospitals, and 215 (70.3%) worked in hospitals with CLP services. Nearly half of the respondents had junior titles (doctors’ titles are typically divided into three levels in China, that is, junior-, mid- and senior-level, which is approximately corresponding to resident, attending and chief physician, respectively), and slightly more than half were physicians. Demographic characteristics and occupational status of respondents are listed in Table 2.

**Table 2:** Items of the questionnaire and overall percentages in agreement

Statement (Code)	Percentage in agreement
General attitude to the management of psychological problems	
Psychological factors are important in the course of physical illness (Q1)	99.3
General Hospital doctors are NOT responsible for emotional care of patients (Q2)	14.4
Dealing with patients’ emotional problems is part of general hospital doctors’ work (Q3)	96.7
I should concern myself with emotional care of regular attenders with chronic physical illnesses (Q4)	94.8
The variety of patients’ emotional and social care enhances my job interest (Q5)	53.9
Emotional care of patients by general hospital doctors is IMPRACTICAL under present conditions (Q6)	52.0
Attitude to assessment and treatment	
Psychological and social factors should be routinely assessed and recorded for inpatients (Q7)	92.8
Psychological and social factors should be routinely assessed and recorded for outpatients (Q8)	70.9
General Hospital doctors should be able to use psychological methods like listening or reassurance (Q9)	98.4
General Hospital doctors should be able to use psychological methods such as discussion of anxiety (Q10)	82.7
General Hospital doctors should be able to prescribe psychotropic drug treatments (Q11)	69.3
When psychological factors appear to be an important cause of the presenting problem, I confine myself to physical assessment (Q12)	27.8
Attitude to psychiatric service and referral	
I would like more contact with the psychiatric service (Q13)	94.1
I would consider referring a patient with *Depression* to psychiatrists (Q14)	92.8
I would consider referring a patient with *Disturbed Behavior* to psychiatrists (Q15)	93.8
I would consider referring a patient with *Diagnostic Problems* to psychiatrists (Q16)	67.6
I would consider referring a patient with *Treatment Non-compliance* to psychiatrists (Q17)	48.0
I would consider referring a patient with *Dementia* to psychiatrists (Q18)	65.4
I would consider referring a patient in *Acute Confusional State* to psychiatrists (Q19)	94.8
Needs in practice	
I would welcome more time to talk to my patients (Q20)	91.5
I would like more help in providing psychological and social care (Q21)	98.7
I would welcome more non medical help in the management of patients’ emotional problems (Q22)	93.1

### General attitudes

All but two respondents (99.3%) agreed that psychological factors play an important role in the course of physical illness; 96.7% thought dealing with patients’ emotional problems was part of general hospital doctors’ work, and 85.6% held the view that general hospital doctors were responsible for emotional care of patients. However, nearly half of the respondents did not think the variety of patients’ emotional and social care enhanced the interest of their work, and more than half held the view that emotional care of patients by general hospital doctors was impractical under current conditions (Table 1). Differences among subgroups divided by demographic characteristics or vocational statuses were further analyzed (Table 3). There were no significant differences detected in most of the comparisons. Educational levels and hospital levels, the respondents worked in, had hardly any effect on attitudes. Female doctors more often held the opinion that they should be concerned with the emotional care of regular attenders with chronic physical illnesses than male doctors (*χ* ^2^ = 6.458, *P* = 0.011), and doctors with middle level title reported least interest in patients’ emotional and social care (vs. Junior title: Wald *χ* ^2^ = 4.647, *P* = 0.031; vs. Senior title: Wald *χ* ^2^ = 5.347, *P* = 0.021). In comparison with physicians, more surgeons and other doctors thought that emotional care of patients was impractical currently (Wald *χ* ^2^ = 3.871, *P* = 0.049 and Wald *χ* ^2^ = 7.510, *P* = 0.006, respectively). It is noteworthy that respondents from hospitals with CLP services were less likely to endorse their responsibility for patients’ emotional care (*χ* ^2^ = 6.377, *P* = 0.012) (Table 3).

**Table 3:** Comparisons among different categories

Categories		Codes of questions (percentage in agreement)																					
		Q1	Q2	Q3	Q4	Q5	Q6	Q7	Q8	Q9	Q10	Q11	Q12	Q13	Q14	Q15	Q16	Q17	Q18	Q19	Q20	Q21	Q22
Gender	Male (*N* = 154)	98.7	18.2	96.1	91.6	58.4	51.9	90.9	66.2	99.4	81.8	72.7	33.1	94.8	92.9	94.8	68.8	51.9	67.5	94.2	92.2	98.7	94.2
	Female (*N* = 152)	100	10.5	97.4	98.0^#^	49.3	52.0	94.7	75.7	97.4	83.6	65.8	22.4^#^	93.4	92.8	92.8	66.4	44.1	63.2	95.4	90.8	98.7	92.1
Age band (years)	<30 (*N* = 127)	100	16.5	95.3	92.9	57.5	48.8	90.6	69.3	97.6	81.9	71.7	27.6	94.5	94.5	95.3	65.4	44.1^$^	66.1	91.3	92.1	98.4	91.3
	30–39 (*N* = 118)	98.3	13.6	98.3	98.3	47.5	58.5	94.1	72.0	99.2	84.7	67.8	28.0	93.2	92.4	91.5	73.7	44.1^$^	64.4	97.5	89.8	99.2	94.1
	≥40 (*N* = 61)	100	11.5	96.7	91.8	59.0	45.9	95.1	72.1	98.4	80.3	67.2	27.9	95.1	90.2	95.1	60.7	63.9^#^	65.6	96.7	93.4	98.4	95.1
Educational level	<Master (*N* = 133)	100	13.5	97.0	96.2	56.4	49.6	92.5	71.4	97.7	86.5	69.2	28.6	92.5	91.0	94.7	64.7	51.1	62.4	94.0	90.2	97.7	92.5
	≥Master (*N* = 173)	98.8	15.0	96.5	93.6	52.0	53.8	93.1	70.5	98.8	79.8	69.4	27.2	95.4	94.2	93.1	69.9	45.7	67.6	95.4	92.5	99.4	93.6
Specialty	Physician (*N* = 163)	99.4	11.0	97.5	96.3	57.7	45.4	93.9	73.6	98.8	82.8	74.2	22.1	95.1	92.0	94.5	71.2	48.5	63.2	95.1	93.9	99.4	94.5
	Surgeon (*N* = 75)	98.7	20.0	96.0	92.0	54.7	58.7	93.3	70.7	100	81.3	66.7	41.3^#$^	92.0	94.7	96.0	64.0	48.0	64.0	96.0	86.7	97.3	92.0
	Other (*N* = 68)	100	16.2	95.6	94.1	44.1	60.3^#^	89.7	64.7	95.6	83.8	60.3	26.5	94.1	92.6	89.7	63.2	47.1	72.1	92.6	91.2	98.5	91.2
Title	Junior (*N* = 144)	100	16.7	95.8	93.1	57.6	51.4	91.0	70.8	97.2	82.6	68.8	27.1	95.1	93.8	94.4	66.0	43.8	65.3	92.4	92.4	98.6	89.6
	Middle (*N* = 103)	98.1	14.6	98.1	98.1	43.7^#$^	58.3	93.2	68.9	99.0	85.4	68.0	31.1	93.2	93.2	93.2	71.8	46.6	68.0	97.1	89.3	99.0	97.1
	Senior (*N* = 59)	100	8.5	96.6	93.2	62.7	42.4	96.6	74.6	100	78.0	72.9	23.7	93.2	89.8	93.2	64.4	61.0	61.0	96.6	93.2	98.3	94.9
Hospital level	<Tertiary (*N* = 83)	100	10.8	97.6	96.4	50.6	54.2	92.8	73.5	97.6	88.0	67.5	21.7	90.4	91.6	91.6	68.7	54.2	61.4	97.6	91.6	98.8	94.0
	Tertiary (*N* = 223)	99.1	15.7	96.4	94.2	55.2	51.1	92.8	70.0	98.7	80.7	70.0	30.0	95.5	93.3	94.6	67.3	45.7	66.8	93.7	91.5	98.7	92.8
CLP service	Yes (*N* = 215)	99.1	17.7	95.8	94.0	55.3	53.0	92.6	68.4	98.1	81.4	70.2	27.4	94.4	94.0	93.5	67.4	43.7	66.5	94.9	90.7	98.6	92.6
	No (*N* = 91)	100	6.6^#^	98.9	96.7	50.5	49.5	93.4	76.9	98.9	85.7	67.0	28.6	93.4	90.1	94.5	68.1	58.2^#^	62.6	94.5	93.4	98.9	94.5

*CLP* consultation-liaison psychiatry

^#^ *P* < 0.05 when compared with the first set in the category

^$^ *P* < 0.05 when compared with the last set in the category

### Attitudes to assessment and treatment

An overwhelming majority (92.8%) of respondents believed that routinely assessing and recording the psychological and social factors was necessary for inpatients, while somewhat fewer (70.9%) thought it was necessary for outpatients (Table 2). Over one-quarter confined themselves to physical assessment even when psychological factors appeared to be an important cause of the presenting problem; male doctors were more likely to be in this category than females (*χ* ^2^ = 4.405, *P* = 0.036) while surgeons were more likely than physicians and other doctors (Wald *χ* ^2^ = 9.940, *P* = 0.002 and Wald *χ* ^2^ = 7.660, *P* = 0.006, respectively). With regard to treatment approaches, most respondents agreed that general hospital doctors should be able to use basic psychological methods such as discussion of anxiety, and about two-thirds considered that they should be able to prescribe psychotropic drugs. No significant between-subgroup differences were observed in response to these questions.

### Attitudes to psychiatric services and referrals

Most respondents would welcome more contact with psychiatric services. When asked about attitudes toward psychiatric referrals, more than 90% respondents reported that they would consider psychiatric referral for patients with depression, disturbed behavior or acute confusion. Fewer, around two-thirds, would consider psychiatric referral for diagnostic uncertainty or dementia, and just under half (48%) would refer patients who are non-compliant with treatment. The main reason for not considering psychiatric referral for dementia was the view that psychiatric treatment is ineffective, many thought dementia should be treated by neurologists. For other conditions, the most common reason for not considering a psychiatric referral was the belief that patients dislike it, followed by the fear of stigmatizing patients by such a referral. Significant between-subgroup difference existed with regard to considering psychiatric referrals for patients with poor treatment compliance. Respondents from hospitals without CLP services (*χ* ^2^ = 5.401, *P* = 0.020) and those aged 40 and above were more likely to consider referrals for these patients (Wald *χ* ^2^ = 6.365, *P* = 0.012 and Wald *χ* ^2^ = 6.235, *P* = 0.013, respectively).

### Attitudes to needs in practice

The responses to questions about the needs in practice were highly consistent. Almost all respondents welcomed more time to communicate with patients and wished to receive more (non-medical) help in psychological and social care, as well as in the management of patients’ psychological problems. These views did not vary between subgroups.

## Discussion

This online survey demonstrates potential widespread positive attitudes toward management of patients’ psychological problems, and indicates a high level of need for mental health services to help manage these problems.

Psychological comorbidity is known to prolong stay in general hospitals [29], increase burden of disease [7], and decrease life satisfaction [30], while timely treatment of psychological/psychiatric issues can enhance patients’ compliance [31], satisfaction [32] and is conducive to the recovery of physical illness [4]. The transformation from the biomedical model to biopsychosocial medical model recognizes the significance of mental health to physical health. However, in clinical practice, patients’ psychological problems are often ignored by general hospital doctors in China. One crucial subjective reason could be non-psychiatric doctors’ negative attitudes toward psychiatry or psychiatric disorders. As reported in a previous survey, Japanese non-psychiatric doctors believed that depression care was beyond the scope of their responsibilities [33], and this could be why respondents reported feeling uncomfortable when managing such patients. Previous studies among Chinese non-psychiatric doctors have reported unsatisfactory recognition rates of common mental disorders [24, 25]. Encouragingly, our study demonstrates that most respondents hold positive attitudes toward psychiatric services and management of patients’ psychological disorders. The vast majority recognized the importance of psychological factors underlying physical illness and endorsed their responsibility for recognizing patients’ emotional problems; even though half thought it was impractical to do so under present conditions and many acknowledged they were not interested in doing this themselves.

A systematic review has provided evidence that CLP services are cost-effective in general hospital settings [34]. Nevertheless, as a review pointed out in 2012, the overall practice of CLP in China remains unsatisfactory and the rates of referrals for psychiatric assessments are very low [17]. In our study, non-psychiatric doctors indicated willingness to liaise with psychiatric services, and majority held positive attitudes towards psychiatric referral, suggesting that the situation in China has improved recently. The major reasons for not considering referring psychiatric referrals were similar to a previous study [22], including apprehension about patient reactions and related stigma. While our respondents favored neurology referral for dementia, psychiatrists can play a role in managing psychological, behavioral and social problems associated with dementia; neurologists and psychiatrists appreciate extensive collaboration [35].

Furthermore, most respondents held the opinion that general hospital doctors should be able to use basic psychological methods and prescribe psychotropic drugs. Also, they thought it was necessary to routinely assess and record psychological and social factors for both inpatients and outpatients. These attitudes differ from the traditional view that psychological problems are mainly the responsibility of mental health practitioners.

Doctors’ attitudes toward management of psychological problems in China have rarely been studied and reported, and thus there are no comparable studies available for estimation of changes over time. However, existing studies on Chinese non-psychiatric doctors’ attitudes toward mental health services in general hospitals [36], as well as a potential increase in recognition rates of depression and anxiety disorders in recent years [18, 19, 37], have suggested a possible improvement in attitudes. These changes would owe much to the implementation of Chinese Mental Health Law in 2013 [27], which requires medical practitioners to pay more attention to patients’ mental status, and encourages timely referrals for suspected psychiatric illness. It is noteworthy that along with the need for skills in managing patients’ psychological distress, lack of time was perceived as a significant barrier for general hospital doctors. This could be related to the widely existing shortage of medical resources in China, similar to in other low- and middle-income countries [38].

In comparison to males, female doctors were less likely to confine themselves to physical assessments when psychological factors were key to the presenting problem. This was similar to the results of a survey conducted in a general hospital in London [22], and might be related to general gender differences in personality traits or social roles [39]. Surgeons were also more likely to limit themselves to physical practice, which could be due to the fact that surgeons are also pressed for time and mainly provide acute care in clinical practice [40]. It is noteworthy that a higher proportion of respondents from hospitals with CLP services felt that they were not responsible for emotional care of their patients, suggesting potential risks of de-skilling and avoidance of clinical responsibility when CLP services are available.

Several limitations need to be taken into consideration and addressed in further studies. Firstly, we performed the survey by means of network platform and mobile application which provided convenience in distribution and participation, but the sample size was relatively small for a nationwide survey. Besides, we cannot calculate the response rate, and confirm the real identities of respondents, due to the nature of an anonymous online survey. Moreover, the coverage was geographically limited, with relatively few respondents were from west and north China. This limitation might be addressed by a more widely known online community for Chinese medical personnel like DingXiangYuan (http://www.dxy.cn/). Secondly, most respondents had high educational levels and worked in tertiary hospitals which represented a higher level of medical care and better medical resources including CLP services, and therefore may have been more likely to be concerned about patients’ mental health. However, larger proportion of doctors in China have lower educational levels and are working in primary and secondary care hospitals where less likely to have CLP services; thus, interpretation of our results should be applied cautiously to these doctors, and similar surveys directed at them are warranted. In addition, social desirability bias [41], the tendency of survey respondents to answer questions in a manner that will be viewed favorably by others, would have affected the results, despite the fact that participation was voluntary and anonymous, and questions in our survey did not point to sensitive topics such as personal incomes, patriotism or illegal acts. Further studies might minimize this risk by optimizing the survey design through methods as reported [42].

## Conclusion

Our pilot study reveals a potential widespread positive attitude towards management of patients’ psychological/psychiatric problems and an urgent need for professional help in managing these by non-psychiatric doctors in China. Further well designed studies with larger samples are warranted to ascertain the needs of more inputs and supports for psychiatric services in general hospitals in China.

## Abbreviations

CLP: Consultation-liaison psychiatry
